# COVID-19 and periodontitis: reflecting on a possible association

**DOI:** 10.1186/s13005-021-00267-1

**Published:** 2021-05-11

**Authors:** Giuseppina Campisi, Maria Eleonora Bizzoca, Lorenzo Lo Muzio

**Affiliations:** 1 Department of Surgical, Oncological and Oral Sciences, University of Palermo, Palermo, Italy; 2 Department of Clinical and Experimental Medicine, University of Foggia, Via Rovelli, 50, 71122 Foggia, Italy; 3 C.I.N.B.O. (Consorzio Interuniversitario Nazionale per la Bio-Oncologia), Chieti, Italy

**Keywords:** Periodontitis, COVID-19, Coronavirus, SARS-CoV-2, IL-6, IL-17

## Abstract

Recent studies have demonstrated a relationship between the severe clinical course of COVID-19 and other chronic diseases such as: cardiovascular disease, hypertension, diabetes mellitus, obesity and chronic renal disease. It may be possible to extend this association to a common and chronic oral disease in adults: periodontitis. Alternatively, the latter could be simply related to the systemic chronic diseases cited above, as already observed in the non-COVID-19 literature. In order to provide an overview and their opinion, the authors in this perspective article will report and discuss the most recent references of interest relating to COVID-19 and periodontitis pathophysiology. Within such a narrative review, the authors will hypothesize that the association between chronic periodontitis and COVID-19 could exist via two pathways: a direct link, through the ACEII and CD147 receptors used by the virus to infect the cells, which would occur in greater numbers in cases of periodontitis (thereby favoring a SARS-CoV-2 infection); and/or an indirect pathway involving the overexpression of inflammatory molecules, especially IL-6 and IL-17. An expression of the latter has been found to play a role in periodontitis, in addition to severe cases of COVID-19, although it is still unclear if it plays a direct role in the worsening of the clinical course.

## Introduction

Coronavirus disease 2019 (COVID-19), due to SARS-CoV-2, is the most pressing and emergent health problem worldwide. While most cases result in mild symptoms (including hyposalivation and an alteration in taste [1]), some cases progress to severe pneumonia and multi-organ failure with the death of the patient, according to the age and the presence of comorbidities [2]. Recent relevant studies have demonstrated the bidirectional association between the severe clinical course of COVID-19 and chronic diseases such as: cardiovascular disease, hypertension, diabetes mellitus, obesity and chronic renal disease [1, 3–5]. In seeking a link between COVID-19 and these chronic diseases, they are garnering worldwide interest in the field of research. This interest may also be extended to include more common, chronic oral diseases in adults, such as periodontitis [6], with already demonstrated correlations and associations with other chronic diseases, as mentioned above [7]. The authors in this perspective article will report the most recent references of interest regarding COVID-19 and periodontal physiopathology in order to improve our understanding of associated or randomly associated factors.

Data from the literature [8–12] has confirmed that the majority of COVID-19 positive patients are male. And male sex was observed to be an independent risk factor, which was associated with refractory disease and death (2.8 % death rate for men vs. 1.7 % for female) [11, 13]. With reference to the hypothesis in this article concerning the association between periodontal pathophysiology and COVID-19, let us begin with the argumentation of the studies showing that SARS-CoV-2 also enters the human body via the oral mucosa [1, 14] through the Angiotensin Converting Enzyme II (ACE2) receptor [15–17], which is highly expressed in the oral mucosa (mostly in the epithelial cells of the tongue) [16, 18] and in the salivary glands [5].

A study by Pascolo et al. has demonstrated the necessary co-expression of ACE2 and trans-membrane serine protease (TMPRSS2), to enable the entry of SARS-CoV-2 into host cells [19]. Indeed, the TMPRSS and furin serve to cleave the virus S protein and thereafter spread the infection [20]. Recently, preliminary data has speculated a new infection route for SARS-CoV-2, in which this virus could use its spike protein to bind to CD147 in order to infect human cells [21]. In support of the hypothesis outlined in this article, this would, therefore, result in a high expression of CD147 in the epithelial cells, particularly those of the oral mucosa [22, 23]. Additional data lending credence to the speculation in this article is that SARS-CoV-2 resides in saliva and nasopharynx in such quantities so as to render swabs with these samples useful for COVID-19 diagnostic tests [24–26]. Furthermore, a recent study by Gupta et al. has demonstrated the presence of SARS-CoV-2 in gingival crevicular fluid (GCF), with a sensitivity of the diagnostic swab of 63.64 % (a salivary swab has a sensitivity of approximately 64 %) [24]. Hence, the fact that SARS-CoV-2 stagnates in the GCF may lead us to conclude that poor oral hygiene may increase the viral load in the oral cavity.

Two possible mechanisms which could explain the association between periodontitis and the COVID-19 disease are: (1) the direct contact of virus with the periodontal tissues, also due to the high expression of ACEII and CD147, as mentioned above; and/or (2) the similar overexpression of several cytokines, a COVID-19 ‘cytokine storm’, with elevated serum levels of IL-1 beta, IL-6, IL-7, IL-10, IL-17, IL-2, IL-8, IL-9, GM-CSF, G-CSF, IFN-gamma, TNF alpha, MIP1A, MIP1B, MCP1 and IP10 [27, 28].

In this regard, the authors of this article have analysed the literature data concerning IL-6 and IL-17, which is also overexpressed in periodontitis [29]. Several studies have suggested the role of IL-6 in the pathogenetic mechanisms of COVID-19 [30–35]: when SARS-CoV-2 infects the respiratory tract, it induces a release of pro-inflammatory cytokines, including interleukin (IL)-1beta and IL-6 [31]. One of the mechanisms making the coronavirus lethal could be the induction of interstitial pneumonia, which is in turn linked to an over-production of IL-6 [34, 36]. Similarly, IL-17 has high levels in the serum of COVID-19 patients [27]. In a recent study, an increase in IL-17, and 14 other cytokines, levels were positively correlated with a higher Murray score for lung injuries [28]. Notwithstanding this, some authors have hypothesized that the role of IL-6 merely serves as a reliable biomarker for the early detection and progression of COVID-19 [37]. Periodontal disease has also been recognized as a cytokine storm-generating disease, especially in the presence of other chronic dysmetabolic diseases, such as diabetes [12, 38–40]. Indeed, diabetes can cause a chronic activation of the immune system with increased levels of circulating leukocytes and pro-inflammatory markers [41]; these include serum levels of inflammation-related biomarkers such as IL-6, C-reactive protein, serum ferritin and the D-dimer coagulation index [42].

## Focus on periodontitis

Periodontitis (i.e. plaque-related periodontal disease) is defined as “a chronic, multifactorial inflammatory disease, associated with dysbiotic plaque biofilms and characterized by the progressive destruction of the tooth-supporting apparatus” [43, 44], and classified by staging and grading systems [45]. The disease of periodontitis is globally widespread: in China it has been estimated that the prevalence of the severe variant is 1.9 %, and that those with diabetes are 2.4 times more likely to possess this variant [46]. In recent years there has been particular interest in probable associations between periodontal infection and systemic diseases [38]. Indeed, periodontal pathogens and their products (including inflammatory mediators such as IL-6) could enter the bloodstream, thereby causing several systemic diseases. Chronic periodontitis has been shown to be a risk factor for: cardiovascular disease, *diabetes mellitus*, respiratory disease, rheumatoid arthritis and other conditions [38]. Indeed, periodontopathic bacteria were found in the bronchoalveolar lavage fluid of patients suffering from pneumonia [47]. In turn, diabetes could increase the risk of developing pneumonia [42, 48, 49] and, thus, the association of periodontitis and diabetes could multiply the risk of pneumonia even in the absence of the SARS-CoV-2 infection [1, 50].

It is of note that periodontitis has a documented, higher prevalence in men (~ 57 %) as compared to women (~ 39 %), thereby signifying a possible sex involvement in disease pathogenesis [51]. Furthermore, there are several microbial exchanges (primarily S. mutans, P. intermedia, F. nucleatum and P. gingivalis) between the oral cavity and the lungs [52, 53], and tooth decay and periodontitis are the oral diseases which are predominantly involved in oral bacterial disequilibrium [54]. In order to explain these exchanges (and, therefore, respiratory infections), two hypotheses have been proposed; (1) cytokines and enzymes produced during a periodontal disease may alter the respiratory epithelium and oral mucosa, thereby favoring infection; and (2) the aspiration of oral pathogens into the lungs [55]. Thus, poor oral hygiene can increase the risk of such mouth-lung exchanges and, therefore, respiratory infections, especially in patients over the age of 70 [56, 57].

A previous study has shown that there was an increase in IL-17-producing cells in the gingival tissue of patients suffering from gingivitis and periodontitis, as compared to healthy controls [58]. The same authors reported have elevated levels of IL-17 in the serum of patients with periodontal disease [58]. The systemic inflammatory burden of periodontitis is also well-documented by virtue of the presence of the marked sensitivity of the C-reactive protein (hsCRP), a component of positive Acute Phase Proteins (APPs), which increase during a phlogistic process [58–60]. Indeed, there is evidence regarding increased levels of chemokine- and cytokine-producing cells in the epithelium of periodontopathic patients, and increased levels of these inflammatory markers in the serum of the same patients [61, 62]. Another positive APP is Galectin-3 (Gal-3), a pro-inflammatory protein which is involved in several mechanisms such as: inflammation, angiogenesis, cell growth, host defence and others [63]. Moreover, elevated levels of Gal-3 are related to severe periodontitis [62]. Finally, a correlation between Gal-3 and SARS-CoV-2 was observed in a recent study in which the morphology of Gal-3 was observed to be similar to the SARS-CoV-2 spike protein [64–67].

## Perspective hypothesis: is there any association between COVID-19 and periodontitis?

Basing our hypotheses on the most recent literature and, therefore, on that which has been definitively demonstrated for periodontitis, several events would seem to correlate with the clinical behavior of COVID-19 with a patient’s periodontal status. By causing ulceration of the gingival epithelium, periodontitis could reduce the protective function of the oral epithelial cells, thereby exposing the patients to an elevated risk of invasion by SARS-CoV-2 [1]. Concurrently, ACE2, TMPRSS2 and furin, which are expressed in the aforementioned oral epithelial cells and the proteases produced by periodontopathic bacteria, could cleave the protein S of the virus, thereby favoring infection. It, therefore, follows that the presence of periodontopathic bacteria could increase the risk of SARS-CoV-2 infection [68].

Various studies have shown that the oral, subgingival-component epithelial cells of periodontal pockets express high levels of CD147 [22, 23] and thus, periodontitis could facilitate SARS-CoV-2 infection through the CD147 route.

Takahashi Y et al., have argued (with their unpublished data) that, when periodontopathic bacteria are aspirated into the lungs, the expression of ACE2 increases in the bronchus and the lungs (also in the oral cavity) due to bacterial and pathogenic factors, such as endotoxins, and that this overexpression could increase the risk of a SARS-CoV-2 infection [68].

In a recent study by Takahashi Y et al., the culture supernatant of the periodontopathic bacterium Fusobacterium nucleatum (CSF) has been shown to upregulate the angiotensin converting enzyme 2 of the SARS-CoV-2 receptor in alveolar epithelial cells [69]. Furthermore, CSF induced the production of interleukin (IL) -6 and IL-8 by alveolar epithelial cells; CSF also strongly induced the expression of IL-6 and IL-8 by bronchial epithelial cells of pharyngeal epithelial cells. These findings suggest that when patients with mild COVID-19 frequently aspirate periodontopathic bacteria, SARS-CoV-2 infection is promoted, and lower respiratory tract inflammation can become severe in the presence of viral pneumonia [69].

Moreover, Marouf N et al., in a case-control study, demonstrated that periodontitis was associated with higher risk of intensive care unit admission, need for assisted ventilation and death of COVID-19 patients, and with increased blood levels of biomarkers linked to worse disease outcomes [70].

Other researchers have hypothesized that, in a given patient with periodontal disease the periodontal situation during a SARS-CoV-2 infection could be aggravated due to the downregulation of ACE2 and an increase in ACE and Ang II, thereby resulting in the involvement of several pro-inflammatory factors [71, 72]. It is also noteworthy that periodontopathic bacteria have been found in the metagenome of severe COVID-19 patients [73]; indeed, it is commonplace to find bacterial superinfections in patients with severe cases of COVID-19 [74].

It is clear that respiratory viral infections could predispose patients to bacterial superinfections, resulting in increased disease severity and mortality [75]. Indeed, strains of the coronavirus have been shown to improve the adhesion of streptococcus to epithelial cells of the respiratory tract, causing complications such as pneumonia and inflammatory damage in the lungs and consequent inhibition of the clearance of bacteria [75]. Finally, a recent study has demonstrated that intensive periodontal treatment reduced the risk of pneumonia in COVID-19 patients [48].

Albeit indirect, another possible mechanism, which could explain the association between periodontal disease and a severe COVID-19 course, could be the overproduction of phlogistic molecules, such as IL-6 and IL-17, in healthy patients and patients with chronic diseases such as diabetes. It should be considered that periodontitis, independently of any other pathology, raises IL-6. Therefore and irrespective of the concept that IL-6 is the cause of severe cases of COVID-19 or a reliable biomarker for COVID-19 [37], periodontitis could modify the level of IL-6 in COVID-19 patients. It can also be hypothesized that in those COVID-19 patients, also suffering from diabetes and periodontitis, levels of IL-6 in circulation deriving from all three pathologies could be envisaged.

Considering the aforementioned research, it is the considered opinion of the authors of this perspective article that it merits further discussion in encouraging reflection and/or experimental studies in the following fields: an analysis of the association and/or risk of periodontitis in COVID-19/diabetic patients, who are at greater risk of and developing both diseases; and to ascertain whether the use of molecules against the S-protein (and, therefore, against Gal-3, or vice versa) could be useful in disrupting the attachment of the virus to the host cells and indecreasing the production of IL-1 and IL-6.

## Data Availability

Data sharing is not applicable to this article as no datasets were generated or analysed during the current study.
